# Should Cardiologists Be Concerned About Malnutrition in ACS Patients?∗

**DOI:** 10.1016/j.jacadv.2023.100633

**Published:** 2023-09-28

**Authors:** Gianluca Campo, Elisabetta Tonet

**Affiliations:** Cardiology Unit, Azienda Ospedaliero Universitaria of Ferrara, Cona, Ferrara, Italy

**Keywords:** acute coronary syndrome, malnutrition, myocardial infarction, older age, physical performance

**Figure:**
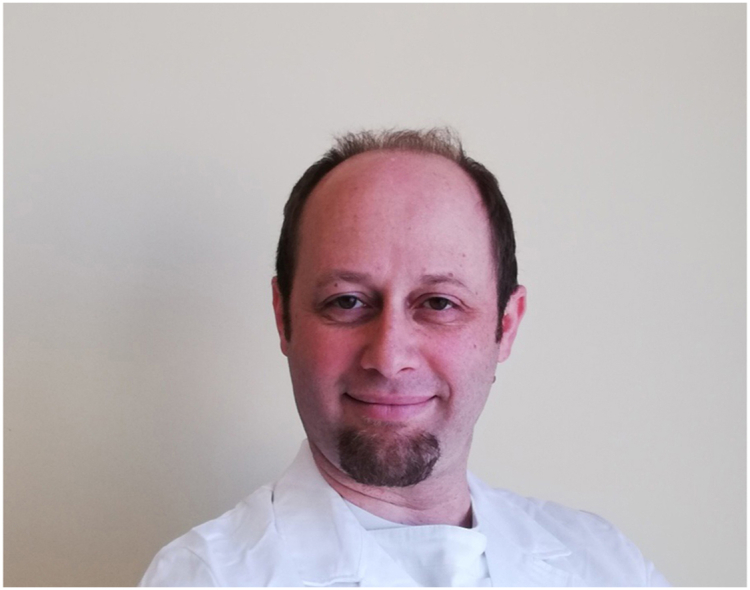

When we talk about malnutrition, the first thing that comes to mind is the poor nutritional status of third-world countries. However, malnutrition also afflicts Western countries and can be seen on our hospital wards. As defined by the World Health Organization, malnutrition is one of the most important threats to public health and corresponds to “a state resulting from lack of intake or uptake of nutrition that leads to altered body composition and body cell mass leading to diminished physical and mental function and impaired clinical outcome from disease”.^1^ Older adults more frequently suffer from malnutrition due to complex and multifactorial reasons.^2^ Aging itself is an established nonmodifiable risk factor for malnutrition; as a matter of fact, higher age is associated with physiological changes such as impaired taste and smell due to an abnormal perception of pleasure associated with food, decreased gastric flexibility, and reduced appetite due to altered gastrointestinal hormones. These factors can slowly result in malnutrition. Additionally, as older age increases the risk for disease, disease itself is a determinant of poor nutritional status.^2^^,^^3^ While this concept may have us think that it is a purely geriatric condition, this is wrong, and cardiologists should be concerned about malnutrition and its negative prognostic impact in all ages.

The aging of the population is associated with a significant increase in the mean age of patients admitted to hospital for acute coronary syndrome (ACS). In the ACS setting, there is a high prevalence of poor nutritional status.^2^ This seems to be due to different mechanisms. Some studies have reported an association between increased levels of inflammation biomarkers such as tumor necrosis factor-alpha, malnutrition status, and poor outcomes in patients with cardiovascular diseases.^4^, ^5^, ^6^, ^7^ Furthermore, the activation of neurohormonal and inflammatory pathways that characterize cardiovascular disease may increase the catabolic demand, so malnourished patients may be more vulnerable to cardiac events. Finally, malnutrition is related to physical decline and limited physical performance, so the beneficial role of physical activity in ischemic cardiac disease is lacking in these patients.^7^ Despite its high prevalence, malnutrition is often overlooked in cardiology department because of a lack of awareness, time, and a universal guideline for the best diagnostic score/tool.

In this issue of *JACC: Advances*, a meta-analysis by Lai et al^8^ is presented. The authors analyzed 30 studies of ACS patients who had undergone a malnutrition assessment. A total of 37,303 individuals were included. The prevalence of malnutrition was impressive, involving approximately one-third of the study population. Comparing the 2 groups of patients (malnourished vs nonmalnourished), malnutrition was significantly associated with all-cause mortality, and the pooled mortality rate in the malnourished group increased year by year reaching 37.9% at 5 years. Interestingly, there was no difference in mortality related to income status or age categories. Regarding the nutritional assessment tools, Lai et al^8^ also found that all included scores had similar prognostic value.

The study highlighted 2 important points. First, it confirmed the adverse prognostic impact of malnutrition in ACS patients, probably related to the genesis and implementation of a proinflammatory status and atherosclerotic burden. Second, it showed the prognostic ability of the currently available screening tools for assessing malnutrition. There is no universal parameter to define malnutrition, but several scores and laboratory parameters have been underdiagnosed. The study by Lai et al^8^ examined 3 nutritional assessment scores and did not find any significant difference in the various scores studied and their relationship with all-cause mortality. This demonstrates that it is not as important which score is used, but rather that malnutrition is identified. As suggested by the authors, the choice of nutritional assessment score should include other considerations such as the ease and availability of prognostic parameters. It is also important to remember that the various tools include different variables to define malnutrition, so that a patient could be considered malnourished based on one score and not with another. Eventually, a universal definition of malnutrition is necessary in order to best plan interventions after myocardial infarction. Additionally, scores need to be administered by trained staff in order to avoid interobserver bias. This point reinforces the need for a universal nutritional assessment score for cardiologist to use in the setting of heart diseases.

As reported above, it is common to find poor nutritional status and/or low physical performance in ACS patients. Recognizing these conditions using specific assessment tools and providing therapeutic strategies to limit disease is of paramount importance. With this background, in recent years, a multidomain approach for cardiac rehabilitation in older people has been promoted. The Finnish Geriatric Intervention Study to Prevent Cognitive Impairment and Disability demonstrated that a multidomain intervention reduced the risk of total cardiovascular events among subjects with a history of cardiovascular disease and among older participants regardless of cardiovascular history. The multidomain program included dietary counseling, exercise training, cognitive training, and management of cardiovascular risk factors. In particular, the dietary intervention was made up of individual counseling and sessions with a nutritionist; the program was based on national dietary recommendations with individually tailored goals for each participant.^9^ The ongoing Physical Activity Intervention in Elderly Patients with Myocardial Infarction Trial is a prospective, randomized, multicenter study focused on a multidomain lifestyle program in older adults after myocardial infarction. It is enrolling patients aged ≥65 years admitted to the hospital for myocardial infarction with low physical performance. The patients are randomized to a multidomain lifestyle intervention vs health education with a composite primary endpoint of cardiovascular death or rehospitalization for cardiovascular causes at 1 year. The multidomain program includes dietary counseling, strict management of cardiovascular and metabolic risk factors, and exercise training. Focusing on dietary intervention, the aim of the program is the identification of malnutrition and education to improve nutritional status. The intervention is tailored for each patient and factors in their nutritional requirements and level of physical performance.^10^

As shown in the Figure 1, cardiologists must move beyond traditional cardiovascular risk factors and begin to consider emerging factors such as malnutrition and poor physical performance. Recognizing these factors and promoting lifestyle intervention (modification of both malnutrition and low physical performance is frequently done concomitantly) is crucial to ensure improvements in prognosis after the acute events.

**Figure 1:**
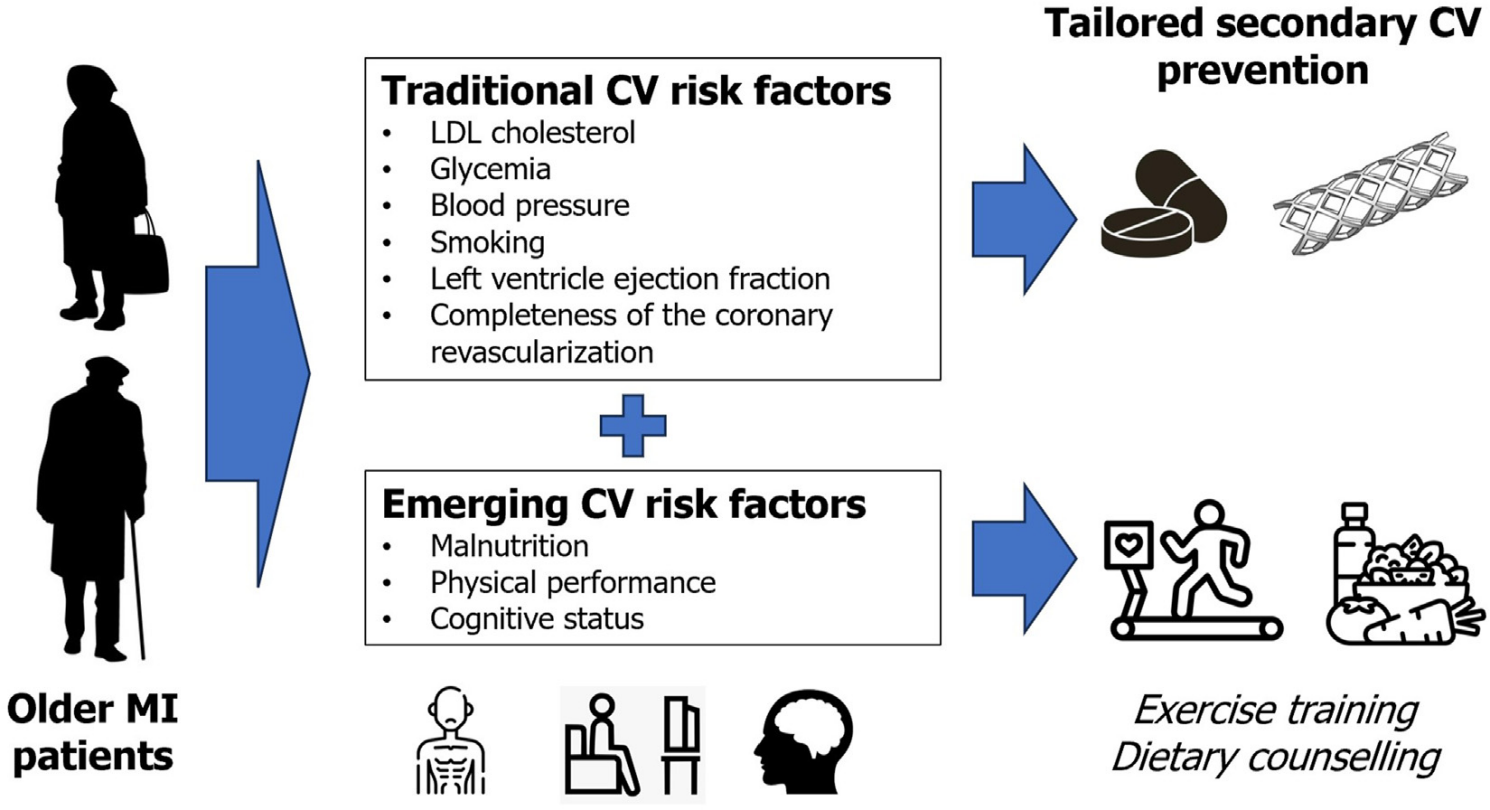
**Malnutrition and Physical Status as Emerging Risk Factors** CV = cardiovascular; LDL = low-density lipoprotein; MI = myocardial infarction.

## Funding support and author disclosures

Dr Campo has received funding from the Italian Health Minister (Ricerca Finalizzata 2018, GR 2018-12367114) for the conduction of the Physical Activity Intervention in Elderly Patients with Myocardial Infarction Trial. Dr Tonet has reported that she has no relationships relevant to the contents of this paper to disclose.
